# Epidemiology, treatment and mortality of trochanteric and subtrochanteric hip fractures: data from the Swedish fracture register

**DOI:** 10.1186/s12891-018-2276-3

**Published:** 2018-10-12

**Authors:** Leif Mattisson, Alicja Bojan, Anders Enocson

**Affiliations:** 1 Department of Clinical Science and Education, Södersjukhuset, Karolinska Institutet. Department of Orthopaedics, Stockholm South General Hospital, 11883 Stockholm, Sweden; 2 Department of Orthopaedics, Sahlgrenska University Hospital Gothenburg/Mölndal, 431 80 Mölndal, Sweden; 3 Department of Molecular Medicine and Surgery, Karolinska University Hospital, Karolinska Institutet, 17176 Stockholm, Sweden

**Keywords:** Hip fracture, Trochanteric fracture, Surgical treatment, Epidemiology, Register study

## Abstract

**Background:**

Hip fractures are a major worldwide public health problem and includes two main types of fractures: the intracapsular (cervical) and the extracapsular (trochanteric and subtrochanteric) fractures. The aim of this study on patients with trochanteric and subtrochanteric hip fractures was to describe the epidemiology, treatment and outcome in terms of mortality within the context of a large register study.

**Methods:**

A descriptive epidemiological register study including patients registered in the national Swedish Fracture Register from January 2014 to December 2016. Inclusion criteria were all primary surgically treated traumatic non-pathological trochanteric and subtrochanteric femoral fractures in patients aged 18 years and above. Individual patient data (age, gender, injury location, injury cause, fracture type, treatment and timing of surgery) were retrieved from the register database. Mortality data was obtained via linkage to the Swedish Death Register.

**Results:**

A total of 10,548 consecutive patients were identified and included in the study. The mean (±SD) age for all patients was 82 ± 11 years and the majority of the patients were females (69%). Most of the fractures were caused by a fall at the same level (83%) at the patients’ accommodation (75%). Fractures were classified using the AO/OTA classification as 31-A1 in 29%, as 31-A2 in 49% and as 31-A3 in 22% of the cases. The most commonly used implant was a short antegrade intramedullary nail (42%), followed by a plate with sliding hip screw (37%). With increasing fracture complexity, the proportion of intramedullary nails was increasing, and also the use of long versus short nails. The majority of the patients were operated within 36 h (90%). There was a higher mortality at 30 days and 1 year for males, and for all those who were delayed to surgery > 36 h.

**Conclusion:**

Safety measures to prevent fall at elderly patient’s accommodation might be a way to reduce the number of trochanteric and subtrochanteric hip fractures. Surgery as soon as possible without delay should be considered to reduce the mortality rate. The selection of surgical methods depends on the fracture complexity.

## Background

Hip fractures are a major public health problem and can lead to disability, reduced quality of life and increased mortality. Hip fractures in general are affecting around 1.5 million people per year worldwide, with the highest rates found in Scandinavia and the lowest in Africa [1, 2]. The number of hip fractures is likely to increase as the number of elderly people is increasing and worldwide it has been estimated that the number of hip fractures will rise to 2.6 million by 2025, and to 6.25 million in 2050 [1, 3]. The hip fractures are a heterogenous group with two main types of fractures: the intracapsular (cervical) and the extracapsular (trochanteric and subtrochanteric) fractures.

The absolute majority of the trochanteric and subtrochanteric hip fracture patients are fragile patients with a tendency to fall and an increased risk of major morbidity and mortality [4–7]. It is important to provide adequate management to these patients and the treatment of choice is normally surgical with internal fixation. The surgical options for these fractures commonly include plating with sliding hip screw or intramedullary nailing, with nailing today being the predominant procedure in many parts of the world [8, 9].

Although most of the authors in the literature recommend that hip fracture patients should be operated without delay to allow early mobilization and thereby reducing mortality and morbidity, the topic is still controversial and the effect of the time to surgery on mortality is debated [10–13].

The Swedish Fracture Register (SFR) is a nationwide register in which data on fracture epidemiology is reported. Previous publications from the register on specific fractures includes humeral and clavicle fractures [14, 15].

The aim of this study on patients with trochanteric and subtrochanteric hip fractures was to describe the epidemiology, treatment and outcome in terms of mortality within the context of a large register study.

## Methods

The SFR started in 2011 and collects information on fractures in extremities, pelvis and spine. The database is national and the data is registered locally at the affiliated departments. Detailed data on fracture epidemiology and treatment is registered and all fractures are registered regardless of treatment type – surgical or non-surgical. To be included in the register the patients need to have a permanent Swedish personal identity number and only fractures that have occurred in Sweden are registered. At the start of this study in January 2014 the number of affiliated departments was 24, and end of the study in December 2016 the number of affiliated departments was 39. The total number of departments in Sweden that are treating fractures is estimated to 54. Data on patient mortality is obtained to the register via linkage to the national Swedish Death Register.

Inclusion criteria in this study were primary surgically treated traumatic non-pathological trochanteric and subtrochanteric femoral fractures in patients aged 18 years and above, occurring from January 2014 to December 2016.

### Variables

Epidemiologic data (patient age and gender), injury data (injury location, cause and date), fracture data (fracture type, treatment and timing of surgery) and mortality data were retrieved from the register database.

Injury location was categorized as: at the patients’ residence or accommodation, in a public place, in a street/road or in an unspecified place. The cause of injury was divided into: a fall at the same level, an unspecified fall, a fall from height, a traffic injury or any other cause. There is no strict guideline for classification of the energy level in the SFR, and it is up to the registering doctor to distinguish between high- and low-energy trauma mechanism. Fractures were classified according to the AO/OTA classification [16] and the ICD-10 code. Surgical implants were categorized as: a short or long antegrade intramedullary nail, a retrograde intramedullary nail, a plate with sliding hip screw, any other type of plate fixation, a hip arthroplasty or any other type of implant. The experience of the main surgeon was divided into: a specialist in orthopaedic surgery, an orthopaedic registrar or any other surgeon. Starting in early 2015, the time of the radiograph confirming the fracture and the time of the start of the operation was included in the register. From these variables, the time (in hours) from the radiograph to the start of the surgery was calculated.

Patient mortality was presented as 30-day and 1-year mortality.

### Statistics

Because of the descriptive nature of the study, statistical testing of the variables was not performed. Variables are presented as proportions of all registered fractures, meaning the available number of inputs in the register excluding any missing values. For scale variables mean ± standard deviations (SD) are presented. The statistical software used was IBM SPSS Statistics, Version 23 for Windows (SPSS Inc., Chicago, USA).

## Results

### Patient and fracture epidemiology

**Fig. 1:**
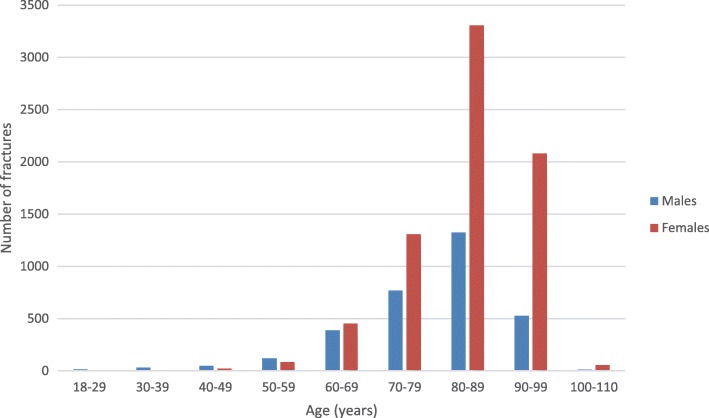
Distribution of trochanteric and subtrochanteric femoral fractures by age and gender

A total of 10,548 entries fulfilling the inclusion criteria were identified in the register and included in the study. The majority of the patients were female (69.4%, *n* = 7317/10548) and the mean (±SD) age for all patients was 82.4 ± 10.5 years. The mean (±SD) age was higher for females (83.9 ± 9.1 years) compared to males (78.9 ± 12.3 years) (Fig. 1).

**Fig. 2:**
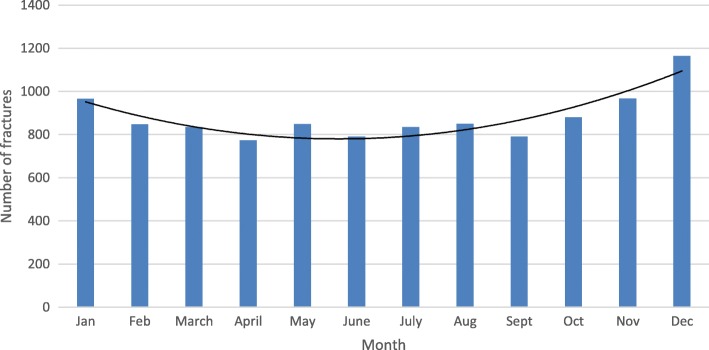
Monthly distribution of trochanteric and subtrochanteric femoral fractures

The location for the trauma was at the patients’ current residence or accommodation in 75% (*n* = 7631/10249) of the cases, in a public place in 4.1% (*n* = 418/10249), in a street/road in 3.7% (*n* = 378/10249) or in an unspecified place in 18% (*n* = 1822/10249). The cause of the injury was a fall at the same level (83%, *n* = 8796/10548), an unspecified fall (9.8%, *n* = 1034/10548), a fall from height (4.0%, *n* = 420/10548), a traffic injury (2.0%, *n* = 212/10548) or any other cause (0.8%, *n* = 86/10548). Fractures were most common during the winter months of January and December (Fig. 2).

**Fig. 3:**
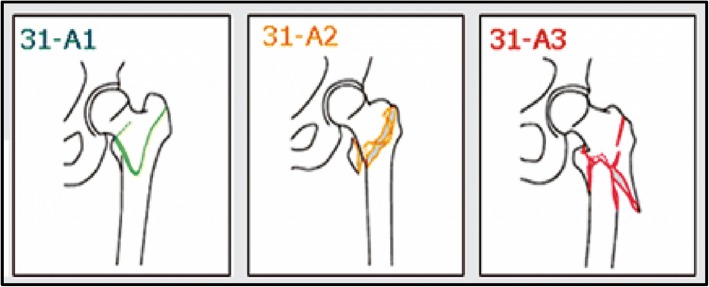
Fracture groups as presented in the SFR. According to AO/OTA classification of trochanteric and subtrochanteric femoral fractures. The use of the figure in this study has been approved by the SFR: 31-A1 Simple trochanteric fractures. 31-A2 Multifragmented trochanteric fractures. 31-A3 Trochanteric reverse oblique and subtrochanteric fractures.

The fractures were classified according to the ICD-10 code system as trochanteric (S72.1) in 78% (*n* = 8260/10548) and as subtrochanteric (S72.2) in 22% (*n* = 2288/10548) of the cases. In addition, fractures were classified using the AO/OTA classification as 31-A1 in 29% (*n* = 3067/10546), as 31-A2 in 49% (*n* = 5191/10546) and as 31-A3 in 22% (*n* = 2288/10546) of the cases (Fig. 3). The fractured side was equally distributed with 50% (*n* = 5253/10548) involving the right side and 50% (*n* = 5295/10548) involving the left side. Fourteen patients (out of 10,548, 0.1%) had an open fracture and 1.6% (*n* = 169/10246) of the fractures were due to a high-energy trauma. Patient and injury epidemiology data in relation to fracture type are presented in Table 1.

**Table 1:** Overview of patient and injury epidemiology in relation to fracture type^a^

	31-A1	31-A2	31-A3
	(*n* = 3067)	(*n* = 5191)	(*n* = 2288)
Age years, mean (±SD)	82.2 (10.3)	83.2 (9.8)	81.0 (12.0)
% (*n*=)			
Female gender	65 (1981/3067)	72 (3725/5191)	70 (1610/2288)
Injury location			
At residence	74 (2202/2960)	75 (3816/5069)	73 (1611/2218)
Public place	4.8 (143/2960)	4.6 (233/5069)	4.4 (97/2218)
Street/road	3.5 (103/2960)	3.6 (183/5069)	4.1 (92/2218)
Other	17 (512/2960)	17 (837/5069)	19 (418/2218)
Injury mechanism			
Fall at same level	84 (2561/3067)	84 (4363/5191)	82 (1870/2288)
Unspecified fall	9.7 (299/3067)	10 (532/5191)	8.9 (203/2288)
Fall from height	3.6 (111/3067)	3.4 (179/5191)	5.7 (130/2288)
Traffic	2.4 (74/3067)	1.7 (87/5191)	2.2 (51/2288)
Other	0.7 (22/3067)	0.6 (30/5191)	1.5 (34/2288)
High energy trauma			
Yes	1.4 (43/2971)	1.1 (55/5045)	3.2 (71/2229)
No	97 (2890/2971)	98 (4924/5045)	96 (2134/2229)
Unknown	1.3 (38/2971)	1.3 (66/5045)	1.1 (24/2229)
Open fracture	0 (0)	0.1 (5/5191)	0.4 (9/2288)

Data is presented in relation to the number of available inputs in the register

^a^Fracture type according to the AO/OTA classification:

31-A1 Simple trochanteric fractures

31-A2 Multifragmented trochanteric fractures

31-A3 Trochanteric reverse oblique and subtrochanteric fractures

### Surgical results

**Fig. 4:**
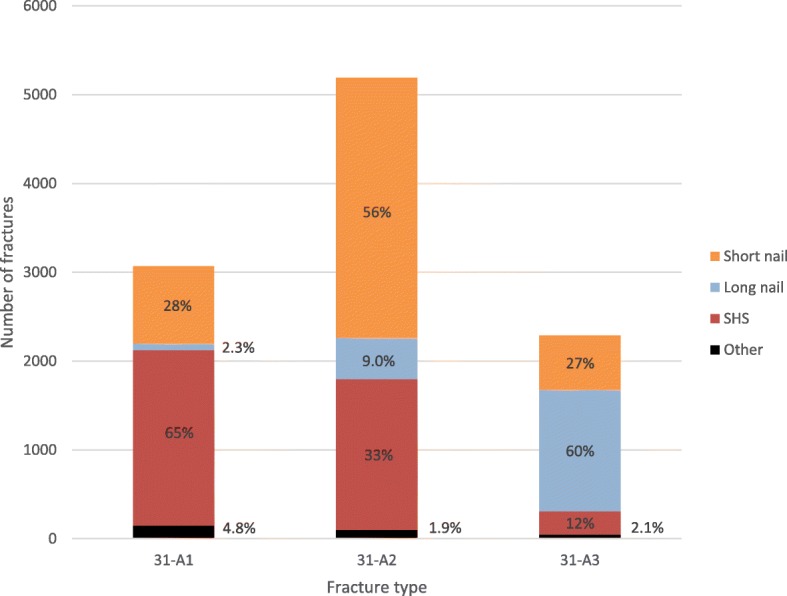
Fracture type according to the AO/OTA classification in relation to treatment

The majority of the patients were operated within 24 h (75%, *n* = 4471/5928), or 36 h (90%, *n* = 5354/5928) from time of the radiograph verifying the fracture to the start of the operation. The operations were performed during night time (22–08 h) in 8.5% (*n* = 522/6126) of the cases. The operations were performed by a specialist in orthopaedic surgery in 62% (*n* = 6348/10186), an orthopaedic registrar in 37% (*n* = 3759/10186) or by any other surgeon in 0.8% (*n* = 79/10186) of the cases. Implants used were: a short antegrade intramedullary nail (42%, *n* = 4411/10548), a plate with sliding hip screw (37%, *n* = 3935/10548), a long antegrade intramedullary nail (18%, *n* = 1903/10548), any other type of plate fixation (1.6%, *n* = 167/10548), a retrograde intramedullary nail (0.6%, *n* = 63/10548), a hip arthroplasty (0.5%, *n* = 58/10548) or other implants (0.1%, *n* = 11/10548). Implants used in relation to fracture type are presented in Fig. 4.

### Mortality

**Fig. 5:**
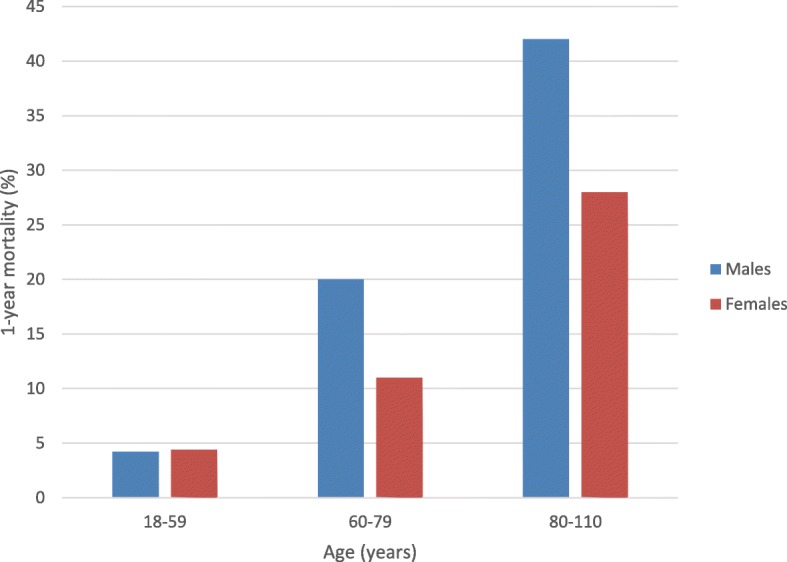
Comparison of 1-year mortality between males and females for different age-groups

The overall 30-day mortality was 7.7% (*n* = 811/10548) and the 1-year mortality was 26% (*n* = 2731/10548). There was a higher 30-day mortality for males (11%, *n* = 355/3231) compared to females (6.2%, *n* = 456/7317). As was there a higher 1-year mortality for males (32%, *n* = 1026/3231) compared to females (23%, *n* = 1705/7317). One-year mortality for different age-groups is presented in Fig. 5. The 30-day mortality was higher for patients operated > 36 h (9.8%, *n* = 56/574) compared to patients operated ≤24 h (7.8%, *n* = 349/4471) or ≤ 36 h (8.0%, *n* = 429/5354). Operations delayed for surgery > 36 h also had a higher 1-year mortality (31%, *n* = 179/574) compared to operations performed ≤24 h (25%, *n* = 1118/4471) or ≤ 36 h (26%, *n* = 1370/5354). Detailed results on mortality are presented in Table 2.

**Table 2:** Mortality in relation to gender, fracture type and surgical factors

	30-day mortality	1-year mortality
	% (*n*=)	% (*n*=)
All	7.7 (811/10548)	26 (2731/10548)
Gender		
Female	6.2 (456/7317)	23 (1705/7317)
Male	11 (355/3231)	32 (1026/3231)
Fracture type^a^		
31-A1	7.2 (222/3067)	26 (793/3067)
31-A2	8.2 (427/5191)	27 (1389/5191)
31-A3	7.1 (162/228)	24 (548/2288)
Time to surgery		
≤ 24 h	7.8 (349/4471)	25 (1118/4471)
≤ 36 h	8.0 (429/5354)	26 (1370/5354)
> 36 h	9.8 (56/574)	31 (179/574)
Time of surgery start		
08–22	8.2 (460/5604)	26 (1476/5604)
22–08	8.0 (42/522)	24 (126/522)
Implant		
Short intramedullary nail	8.2 (362/4411)	27 (1167/4411)
Long intramedullary nail	6.8 (130/1903)	24 (450/1903)
Plate with sliding hip screw	7.6 (298/3935)	26 (1030/3935)
Other	7.0 (21/299)	28 (84/299)

Data is presented in relation to the number of available inputs in the register

^a^Fracture type according to the AO/OTA classification:

31-A1 Simple trochanteric fractures

31-A2 Multifragmented trochanteric fractures

31-A3 Trochanteric reverse oblique and subtrochanteric fractures

## Discussion

In this observational study of trochanteric and subtrochanteric hip fractures in Sweden during the years 2014 to 2016 the majority of the patients were women (69%) with a high mean age (84 years) and a fracture caused by a fall at the same level at their accommodation. The most common type of fracture according to the AO/OTA classification was a type 31-A2 and the majority of the patients were operated with a short intramedullary nail within 36 h. There was a higher mortality at 30 days and 1 year for males, and for all those who were delayed to surgery > 36 h.

### Epidemiology

Our finding of a higher number of fractures among females is in line with most of the previous studies and has an association with osteoporosis [4, 17–21]. The high age of the patients reflects that there is an increased risk to fall with advanced age, and as these patients often are frail with poor bone-quality there is an increased risk for suffering from a hip fracture even after a low energy fall. Mangram et al. 2014 [17] described that 73% of their trochanteric fracture patients fell at home. Similarly, Haginoa et al. 2017 [6] reported that an indoors simple fall was the trauma mechanism in 80% of their hip fracture patients, and 85% of them were ≥ 90 years old. Interestingly we found that fractures were slightly more common during the winter months of January and December, despite that the majority of them occurred indoors at the patients’ accommodation. But this finding is in line with previous studies. Pueyo-Sanchez et al. 2017 [22] reported that in Catalonia, Spain a seasonal variation was observed with more cases in the winter. Similarly, Gronskag et al. 2010 [23] found a seasonal variation in hip fracture incidence among elderly women in Norway, which was characterized by higher fracture rates during the winter months. Finally, Hagino et al. 2017 [6] reported a monthly variation were January had the highest number of patients per month during the observation period.

### Fracture and treatment characteristics

We found that the AO/OTA type 31-A1 and 31-A2 fractures, which are sometimes referred to as stable, were most common (78%). This finding is similar to Chehade et al. [20] who in 2015 published a prospective consecutive cohort of 743 patients were the majority (60%) were classified as stable trochanteric and only 40% as unstable trochanteric or subtrochanteric fractures. We found that with increasing fracture complexity, the proportion of intramedullary nails was increasing, and also the use of long versus short nails. Previous literature have advocated superiority of intramedullary nails, compared to plate with sliding hip screw, and proposed advantages in the management of unstable trochanteric and subtrochanteric fractures in providing biomechanical stability and improved functional outcome [8, 9, 24–27]. Furthermore, a long nail can offer protection all along the femur, in comparison to a short nail [28–30]. In our observational study we found that although intramedullary nailing was the most commonly used implant overall, the plate with sliding hip screw was commonly used as well, especially for the AO/OTA type 31-A1 fractures (Fig. 4). The “plate versus nail” debate is probably not yet over as the latest Cochrane report comparing intramedullary nails with plate and sliding hip screw concluded that “sliding hip screw appears superior for trochanteric fractures” [31].

### Mortality

We found a higher mortality rate in males, despite younger mean age. As a comparison, the expected 1-year mortality for an unselected population of 80 years old in Sweden 2017 was 3.5% for females and 4.8% for males (www.statistikdatabasen.scb.se). In 2010 Kannegaard et al. [32] observed in a nationwide register-based cohort study including more than 41,000 Danish hip fracture patients, increased 1-year mortality in men and that the mean survival time was slightly shorter after trochanteric and subtrochanteric fracture (3.3–3.4 years) compared with other types of hip fractures (3.5–3.8 years). Haentjens et al. [33] performed time-to-event meta-analyses and showed that the relative hazard for all-cause mortality in the first 3 months after a hip fracture was 5.75 in women and 7.95 in men. The majority of our population was operated within 24 h (75%) or 36 h (90%) calculated from the time of the radiograph verifying the fracture to the start of the operation. This is consistent with the current recommendations for the management of hip fractures in many settings, although we still lack an international consensus. Obviously, several factors can affect the postoperative mortality, but the time to surgery is one of the most debated ones. Moja et al. 2012 [34] described in a meta-analysis that a delay to surgery was associated with a significant increase in the risk of death and pressure sores, and recommended that most patients with a hip fracture should be operated within 1 or 2 days. In addition, early fracture fixation and mobilization of these patients decreases the economic burden as it might reduce the overall length of stay, and thus the total cost [35]. On the contrary, a recent prospective cohort study from Lizaur-Utrilla et al. 2018 including 1234 patients who underwent hip fracture surgery suggested that waiting time for the surgery more than 2 days to stabilize patients with active comorbidities at admission was not associated with higher complication or mortality rate. However, the patients who were delayed to surgery due to organizational reasons had a significant higher rate of postoperative complications and 1-year mortality [36]. To be able to operate patients within 24 or 36 h one might need to operate also at nighttime (22:00–8:00 h). We found no relation between mortality and the starting time of the surgery, whether it was performed during daytime or nighttime. Although other studies have defined the nighttime slightly different (16:00–07:00 h) they showed similar results [37, 38].

### Strengths of the study

The major strength of this study is the large number of included fractures. The data from the SFR provides prospective data on a national level regardless of local differences in epidemiology, socio-demographics and treatment traditions. The included data is detailed and the register have been well validated [39–41]. Another strength is the mortality data that provides a unique opportunity to integrate epidemiologic data with a relevant outcome measurement.

### Limitations of the study

The SFRs coverage during the study period (January 2014 to December 2016) included with increasing number of participating departments by the end of 2016 approximately only 72% of Sweden’s orthopaedic departments. However, the remaining clinics that have not yet signed up are mostly smaller units, so in reality the national proportion of excluded fractures is most likely small, but still the incomplete coverage of the SFR as a limitation. Registration of the time for the radiograph confirming the fracture and the start of the operation did not start until 2015. The number of valid inputs in the register on this topic is therefore somewhat limited, but since the total number of valid inputs is large, we still think that the results regarding delay to surgery and timing of the operations are valid indicators and represents true national trends. Another limitation is that due to the descriptive nature of this register study all the results were unadjusted regarding different reasons for delay to surgery, implant choice or co-morbidities of the patients.

## Conclusion

Safety measures to prevent fall at elderly patient’s accommodation might be a way to reduce the number of trochanteric and subtrochanteric hip fractures. Surgery as soon as possible without delay should be considered to reduce the mortality rate. The selection of surgical methods depends on the fracture complexity.

## Abbreviations

AO: Arbeitsgemeinschaft fur Osteosynthesefragen
OTA: Orthopaedic Trauma Association
SFR: Swedish Fracture Register
